# Supplementary material for: Emotional disclosure as a therapeutic intervention in palliative care: a scoping review protocol
Source: BMJ Open. 2019 Aug 26;9(8):e031046. doi: 10.1136/bmjopen-2019-031046 (PMC6720334; doi:10.1136/bmjopen-2019-031046)
Supplement: Supplementary data [file bmjopen-2019-031046supp002.pdf]

Supplementary file 2. Search strategy for Ovid PsycINFO: 1806 to 2019

1 exp Emotions/ (300490)
2 emotion* (382265)
3 feeling* (103050)
4 1 OR 2 OR 3 (598583)

****

5 Palliative Care/ (11016)
6 (palliat* or terminal* or endstage or hospice* or metasta* or (end adj3 life) or (care adj3 dying) or ((advanced or late or last or end or final) adj3 (stage* or phase*))).tw. (54673)
7 5 OR 6 (54847)

****

8 (disclos* or express* or communicat* or talk* or speak* or spoke* or writ* or draw* or sing*).mp. (1005983)

****

9 4 AND 7 AND 8 (2575)

****

10 Apply filter: Humans (2278)
11 Apply filter: Adulthood (18+ years) (1320)
