# Supplementary material for: Emotional disclosure as a therapeutic intervention in palliative care: a scoping review protocol
Source: BMJ Open. 2019 Aug 26;9(8):e031046. doi: 10.1136/bmjopen-2019-031046 (PMC6720334; doi:10.1136/bmjopen-2019-031046)
Supplement: Supplementary data [file bmjopen-2019-031046supp003.pdf]

Supplementary file 3. Data charting extraction categories

Citation (Authors, year, title, journal, volume, page):

Keywords:

1. Population

a. Country:
b. Size (n):
   a. Total
   b. Intervention
   c. Control group(s)
c. Disease and stage
d. Age
e. Sex
f. Ethnicity
g. Other of note
h. Patient healthcare setting (hospice, hospital etc.)

2. Study details

i. Study design
j. Study objective

3. Intervention details

k. Intervention name
l. Objective of intervention
m. Task description
n. Control task description
o. Number of task sessions total
p. Task schedule (days between tasks)
q. Intervention setting (where task is completed)

4. Outcome measures

r. Primary
s. Secondary
t. Other measures (covariates, moderators)
u. Assessment time-points

5. Results

v. Primary outcome results
w. Secondary outcome results
x. Other results

6. Setting specific intervention details

y. Explicit adaptations to the intervention
z. Facilitators and barriers to intervention feasibility

7. Theoretical frameworks described
