# Supplementary material for: Emotional disclosure as a therapeutic intervention in palliative care: a scoping review protocol
Source: BMJ Open. 2019 Aug 26;9(8):e031046. doi: 10.1136/bmjopen-2019-031046 (PMC6720334; doi:10.1136/bmjopen-2019-031046)
Supplement: Author's manuscript [file bmjopen-2019-031046.draft_revisions.pdf]

BMJ Open is committed to open peer review. As part of this commitment we make the peer review history of every article we publish publicly available.

When an article is published we post the peer reviewers' comments and the authors' responses online. We also post the versions of the paper that were used during peer review. These are the versions that the peer review comments apply to.

The versions of the paper that follow are the versions that were submitted during the peer review process. They are not the versions of record or the final published versions. They should not be cited or distributed as the published version of this manuscript.

BMJ Open is an open access journal and the full, final, typeset and author-corrected version of record of the manuscript is available on our site with no access controls, subscription charges or pay-per-view fees (http://bmjopen.bmj.com).

If you have any questions on BMJ Open's open peer review process please email info.bmjopen@bmj.com

BMJ Open

Emotional disclosure as a therapeutic intervention in palliative care: a scoping review protocol

Journal:	*BMJ Open*
Manuscript ID	bmjopen-2019-031046
Article Type:	Protocol
Date Submitted by the Author:	12-Apr-2019
Complete List of Authors:	McInnerney, Daisy; University College London, Marie Curie Palliative Care Research Department<br>Kupeli, Nuriye; University College London, Marie Curie Palliative Care Research Department<br>Stone, Patrick; University College London, Marie Curie Palliative Care Research Unit<br>Anantapong, Kanthee; University College London, Marie Curie Palliative Care Research Department; Prince of Songkla University Faculty of Medicine, Department of Psychiatry<br>Chan, Justin; University College London, Marie Curie Palliative Care Research Department<br>Candy, Bridget; University College London, Marie Curie Palliative Care Research Department
Keywords:	Emotional disclosure, Expressive writing, Psychotherapy, Advanced disease, PALLIATIVE CARE

SCHOLARONE™
Manuscripts

TITLE

Emotional disclosure as a therapeutic intervention in palliative care: a scoping review protocol

AUTHORS

Daisy McInnerney[1] (corresponding author), Marie Curie Palliative Care Research Department, Division of Psychiatry, University College London, London, UK; ORCID ID: 0000-0002-8921-2215

Nuriye Kupeli, Marie Curie Palliative Care Research Department, Division of Psychiatry, University College London, London, UK; ORCID ID: 0000-0001-6511-412X; n.kupeli@ucl.ac.uk

Patrick Stone, Marie Curie Palliative Care Research Department, Division of Psychiatry, University College London, London, UK; ORCID ID: 0000-0002-5765-9047; p.stone@ucl.ac.uk

Kanthee Anantapong, Marie Curie Palliative Care Research Department, Division of Psychiatry, University College London, London, UK and Department of Psychiatry, Faculty of Medicine, Prince of Songkla University, Thailand; kanthee.anantapong.18@ucl.ac.uk

Justin Chan, Marie Curie Palliative Care Research Department, Division of Psychiatry, University College London, London, UK; ORCID ID: 0000-0003-4144-441X; uctvjec@ucl.ac.uk

Bridget Candy, Marie Curie Palliative Care Research Department, Division of Psychiatry, University College London, London, UK; b.candy@ucl.ac.uk

[1] Marie Curie Palliative Care Research Department, Division of Psychiatry, University College London, 6th Floor, Maple House, 149 Tottenham Court Road, London W1T 7NF; Email: daisy.mcinnerney.18@ucl.ac.uk; Phone: +44(0) 7833 296 035

For peer review only - http://bmjopen.bmj.com/site/about/guidelines.xhtml

WORD COUNT

3879

For peer review only

For peer review only - http://bmjopen.bmj.com/site/about/guidelines.xhtml

ABSTRACT

Introduction

Emotional disclosure (ED) is a term used to describe the psychotherapeutic expression of emotion. ED underlies a variety of therapies aimed at improving wellbeing for various populations, including people with palliative-stage disease and their family carers. Systematic reviews of ED-based psychotherapy have largely focused on expressive writing (EW) as a way of generating ED. However, heterogeneity in intervention format and outcome measures has made it difficult to analyse efficacy. There is also debate about the mechanisms proposed to explain the potential effects of ED.

We present a scoping review protocol to develop a taxonomy of ED-based interventions to identify and categorise the spectrum of interventions that could be classified under the umbrella term of 'emotional disclosure' in the palliative care setting. By mapping these to associated treatment objectives, outcome measures and explanatory frameworks, the review will inform future efforts to design and evaluate ED-based therapies in this population.

Methods and analysis

The review will be guided by Arksey and O'Malley's five-stage scoping review framework and Levac's extension. The following electronic databases will be searched from database inception: CENTRAL, CINAHL, PsycINFO, Scopus, Web of Science and MEDLINE. We will include peer-reviewed studies and reviews. We will also check grey literature, including clinical trial registers, conference proceedings and reference lists, as well as contacting researchers. Articles will be screened by at least two independent reviewers and data charted using an extraction form developed for this review. Results will be analysed thematically to create a taxonomy of interventions, outcome measures and theoretical frameworks.

Ethics and dissemination

For peer review only - http://bmjopen.bmj.com/site/about/guidelines.xhtml

This review does not require ethical approval as it is a secondary analysis of pre-existing, published data. The results will inform future research in the development of ED-based interventions and evaluation of their efficacy in the palliative care setting. We will disseminate findings through peer-reviewed journals.

ARTICLE SUMMARY

Strengths and limitations of this study

- Despite the heterogeneity of the intervention repeatedly being cited as a limiting factor in evaluations of intervention efficacy, no previous study that we are aware of has systematically mapped the variety of ED-based interventions, outcome measures and theoretical frameworks used in the palliative care setting.
- A rigorous, systematic approach will be applied to searching, screening, extracting and analysing the literature based on established scoping review methodological approaches.
- There may be challenges in identifying studies due to the differences in terms used to describe ED-based interventions.
- The review will be limited to studies of adult populations and published in the English language.
- The studies included in the review will not be appraised for methodological quality as this is outside the remit of scoping review methodology.

KEY WORDS

Emotional disclosure; expressive writing; psychotherapy; palliative care; advanced disease

For peer review only

INTRODUCTION

People living with a terminal illness often experience significant psychological, emotional and physical discomfort.(1,2) Family carers are also likely to experience psychological distress during and after supporting a relative through advanced illness.(2–4) Palliative care services aim to holistically address these physical, psychological and other needs of patients with advanced, life-limiting illness.(5) Psychotherapies form one important element of this palliative treatment approach.(6–8) However, access to such therapies in this setting is restricted by issues arising from limited availability of qualified professionals and evidence-based interventions well-adapted to the population.(9,10) Funding also poses a potential challenge, with a 2015 survey of UK hospices identifying significant concerns over freezing or reduction of statutory funding, and warning of its adverse effects on hospice services.(11,12) Furthermore, guidelines for the provision of psychological services in this population are limited. The National Institute of Health and Care Excellence (NICE) last published guidance on structuring psychological support in palliative care for adults in 2004 and only in cancer. It is not clear how widely these guidelines have been followed.(9,13) Indeed, a recent survey of clinical psychologists working in UK hospices indicated there is significant variability in how psychological support is provided.(9) Palliative care physicians also report limited access to psychological services for patients.(14) Taken together, this suggests that palliative care services might benefit from access to simple interventions that can be delivered by non-specialist health care practitioners, and with the potential for volunteer involvement. This could supplement the support offered by specialist practitioners to clinically distressed patients with complex needs.

For peer review only - http://bmjopen.bmj.com/site/about/guidelines.xhtml

Emotional disclosure (ED) is a term used to describe the therapeutic expression of emotions. This flexible therapy holds potential to be harnessed as a relatively low-cost, simple intervention in certain formats, such as expressive writing.(15) Its flexibility also means it could be adapted to the specific needs of patients at a palliative stage of disease, for example, by modifying the method used to generate emotional expression (for example, typing or spoken disclosure). ED has long been a critical concept associated with the talk-based psychotherapies pioneered by Freud in the early 1900s.(15) However, rigorous research into the concept and associated therapies was relatively scant until the 1980s.(15) In 1986, Pennebaker and Beall introduced their influential Expressive Writing (EW) intervention, which was designed to generate ED and led to a rapid expansion of research into this topic.(15,16) EW typically involves participants writing about their emotions associated with a traumatic experience for 15-20 minutes over 3-5 consecutive days. Since the introduction of Pennebaker and Beall's EW intervention, the format has been widely adapted to explore the boundary conditions of the intervention.(16–19) For example, tasks to induce ED have included writing about positive emotions or future goals, or spoken disclosure.(18–23) Poetry therapy is also recognised as an adaptation of the original EW paradigm.(22,24) Moreover, ED is recognised as a fundamental part of other forms of psychotherapy, such as music therapy(25) and art therapy.(26) In recent times, patients and family members are increasingly turning to blogging, social media and chatroom sites to disclose emotions around their experiences of their illness.(27–29)

The existence of numerous formats of ED-based therapies and behaviours complicates the process of exploring if and how such interventions might work. Yet, a clear understanding of the mechanisms linking cause and effect is considered fundamental to the development and study of complex interventions.(30,31) In the case of ED, there is unlikely to be a single underlying process, rather a framework of interacting mechanisms.(19,32,33) Processes that have been proposed to explain EW

For peer review only

For peer review only - http://bmjopen.bmj.com/site/about/guidelines.xhtml

have been reviewed and include emotional inhibition, cognitive adaptation, exposure and emotional regulation,(19,32,33) yet no consensus has been reached on a unifying framework. Different methods of generating ED are also likely to invoke different or overlapping processes. For instance, disclosure through EW may employ mechanisms related to language processing(34–36) and ED through art may function through sensory and motor processes.(37) Disclosure via online forums is likely to involve many of the mechanisms involved in social support.(38–40) Moreover, the therapeutic setting may influence which cognitive processes are initiated by ED. For instance, for patients at the palliative stage of disease, the potential effects of ED may be mediated by mechanisms related to meaning, control or closure in ways that may not occur in a healthy population.(41,42)

Mirroring the unclear processes underlying ED, the efficacy of ED-based therapies remains uncertain. In general populations, a meta-analysis of 146 studies identified a small but significant positive effect of ED-based interventions on both physical and psychological health outcomes in healthy populations.(43) It has been suggested that moderators, such as demographic, personality, and existing emotional support, are also likely to influence the efficacy of ED-based therapies, highlighting the importance of targeting and tailoring such interventions.(43) In people receiving palliative care, evidence of efficacy also remains unclear. Much of the literature is focused on EW and reports mixed results. A recent systematic review and meta-analysis of four randomised controlled trials (RCTs) that examined EW in patients with advanced disease found it did not have a significant effect on any of the selected health-related outcomes.(44) However, the review also reported more promising results from studies that conducted linguistic analyses of EW in this population; these analyses identified that use of certain words, such as positive emotion words, was related to better emotional wellbeing.(45) They also found EW writers used more cognitive words associated with causal understanding, which the authors reported suggested cognitive changes.(46) Other related reviews have also uncovered mixed results. A systematic review of EW in patients with

For peer review only

breast cancer (irrespective of stage) found the intervention reduced negative somatic symptoms at a 3-month follow-up, although it had no effect on psychological outcome measures.(47) Another review(48) assessing EW in a variety of cancers of all stages reported 6 of 13 studies as finding statistically significant, small to moderate benefits of EW on energy and sleep patterns,(49) depressive(50) and physical symptoms,(50,51) emotional support,(52) pain,(53) uptake of mental health services,(54) and healthcare utilisation.(51) However, each of the 13 studies also reported some null effects, and the authors were unable to conduct a meta-analysis due to the heterogeneity of interventions and outcome measures used across the studies. A further review of EW in cancer populations found no evidence of EW efficacy on psychological, physical of quality of life outcomes,(55) whilst a review of therapeutic writing in patients with long-term illnesses also found no effects.(22) None of the studies of EW in populations with advanced disease reported significant negative effects, with the exception of Low et al. (2010), who found women who had been diagnosed with metastatic cancer for ≥ 4.7 years exhibited greater sleep disturbance following EW, whereas those more recently diagnosed did not.

Such mixed results are also characteristic of other ED-based interventions in clinical populations. A preliminary scoping of the literature indicates that reviews of studies of explicitly ED-based interventions in the palliative care setting are lacking, individual studies have identified some benefits in this population. For instance, Kissane et al. (2016) found supportive-expressive group therapy improved quality of life and treatment of depression in women with metastatic breast cancer.(56) Whilst Clements-Cortes (2004) found patients with a terminal illness were able to decrease depressive symptoms and social isolation and enhance relaxation by expressing their emotions through music therapy.(57) Indeed, a recent review of music therapy in patients receiving palliative care found it had a positive effect on pain, fatigue, anxiety and quality of life.(58) Given the heterogeneity of interventions included in the review, however, not all included interventions were necessarily designed specifically to evoke ED. Reviews of ED-based interventions in clinical

For peer review only - http://bmjopen.bmj.com/site/about/guidelines.xhtml

populations with serious (although not explicitly palliative-stage) disease have identified mixed results. A systematic review of 52 trials reported that music interventions may have beneficial effects on anxiety, pain, fatigue and quality-of-life for people with cancer (of all stages), but that results were inconsistent across trials.(59) They also noted that, when asked, participants said they valued the opportunity for emotional expression and processing offered by the therapy. A further systematic review of Creative Psychological Interventions (CPIs), which encompass the use of music, art, drama and dance/movement to express and process thoughts and emotions, found evidence of psychological but not physical benefits of CPIs across ten trials in cancer patients.(60) Similarly, ED-based interventions in bereaved family members have also demonstrated mixed results,(61,62) although they have been less widely studied in this group than in patients.

Whilst the efficacy of ED-based therapies in the palliative care setting remains uncertain, current reviews recommend further research to assess the true efficacy of each intervention.(22,44,47,48,55,59,60) This is due to a number of limitations of the current literature. First, current reviews are significantly limited by the heterogeneity of the format of interventions and outcome measures used across the studies they are reviewing.(22,44,47,48,55,59,60) Second, and tellingly, qualitative interviews show participants find certain interventions valuable, even where null effects are captured by quantitative measures.(48,59) This suggests current studies are not necessarily investigating the outcome measures that convey the benefit experienced by patients, which may be more abstract or existential in nature, particularly in patients with a palliative-stage disease.(48,63) Third, authors have noted a lack of effort to tailor interventions to the specific needs of people with advanced or terminal illness.(22,44,48) This could encompass, for example, offering audio recorded disclosure as an alternative to written disclosure in EW studies, as some patients may lose the ability to write. Finally, methodological quality of studies included in the systematic reviews has been largely graded as low, with limitations due to sample size, methodological features such as lack of randomisation and data reporting. In light of these

For peer review only - http://bmjopen.bmj.com/site/about/guidelines.xhtml

significant limitations to existing research, future studies should aim to address these shortfalls. The broad nature of therapeutic ED, however, makes future research design challenging. This is due, in part, to the significant overlap of terms being used to describe various interventions, and a lack of clarity on the most appropriate format and outcome measures and underlying mechanisms.

Objectives

Through the flexible yet systematic process afforded by scoping review methodology,(64–66) this review will develop a taxonomy of ED-based interventions used in the palliative care setting, for people with advanced diseases and their family carers. The taxonomy will identify, categorise and define classes of intervention that fall under the umbrella term of 'emotional disclosure'. In line with recommendations for complex intervention design, we will also map classes of intervention to underlying mechanisms, appropriate treatment objectives and outcome measures.(30,31) The taxonomy will provide researchers with a framework to inform the design of future studies of ED-based interventions, by guiding selection of intervention format and outcome measures. Moreover, the taxonomy will map intervention efficacy, along with any reported facilitators and barriers, to intervention format, to help draw out potential mechanisms of action. If researchers use the taxonomy to inform study design, this should in turn lay the groundwork for more informative systematic reviews of ED-based interventions. Given the unique physical, psychological and emotional position of patients at the palliative stage of disease and their family carers, the scope of this review will be limited to research conducted in the palliative care setting to ensure the findings are applicable to the population of interest. However, the findings could also provide a springboard for attempts to develop a taxonomy for ED-based interventions in other populations.

For peer review only - http://bmjopen.bmj.com/site/about/guidelines.xhtml

METHODS AND ANALYSIS

The scoping review methodology allows explorative, yet systematic, investigation of the heterogeneous literature on ED-based therapies.(64–66) This protocol is guided by the standard framework proposed by Arksey and O'Malley(65) and expanded by Levac and colleagues(64) and the Joanna Briggs Institute.(66) These guidelines recommend organising the scoping review process into at least five stages, with an optional sixth stage:

- Stage 1. Identifying the research question(s) - complete
- Stage 2. Identifying relevant studies
- Stage 3. Study selection
- Stage 4. Charting the data
- Stage 5. Collating, summarising and reporting the results
- Stage 6. Consultation

The protocol has also been developed in line with scoping review best practice, as summarised in the completed Preferred Reporting Items for Systematic Reviews and Meta-Analyses for Protocols and Scoping Reviews (PRISMA-ScR) included in supplementary file 1. Table 1 summarises the proposed timescale for the review.

	**Month (2019)**									
	Jan	Feb	Mar	Apr	May	Jun	Jul	Aug	Sep	Oct →
**Stage 1.** Identifying research question and writing protocol										
**Stage 2.** Identifying relevant studies (search)										
**Stage 3.** Study selection (screening)										
**Stage 4.** Data charting										
**Stage 5.** Collating,										

For peer review only

summarising and reporting results		
**Stage 6.** Consultation	*Throughout process at key stages (protocol development, screening, collating results)*	

Table 1. Timeline for protocol and scoping review

Stage 1: Identifying the research question

To meet the objectives of the review, as outlined above, we will seek to thematically analyse insights from the following research questions:

- Which psychotherapeutic interventions delivered in patients at the palliative stage of disease and their family carers are categorised as, or explicitly grounded in, principles of ED? E.g. What format are the interventions, how often are they delivered and by whom?
- What are the primary objectives of ED-based interventions delivered in this setting? For example, to enhance overall quality of life, physical or psychological health.
- What outcome measures are used to assess the efficacy of ED-based interventions in this setting?
- What theoretical frameworks are used to explain the mechanisms underlying ED-based interventions in this setting?

Stage 2: Identifying relevant studies

The following inclusion criteria were developed in collaboration with key stakeholders, including physicians, psychologists and supportive care service managers involved in the provision of palliative care. Throughout the screening and data extraction process the criteria will be discussed within the research team and updated where necessary to ensure all relevant literature is being captured.(64)

- **Studies must use or make reference to a psychotherapeutic intervention that the authors state:**

For peer review only - http://bmjopen.bmj.com/site/about/guidelines.xhtml

   - involves ‘emotional disclosure’ or involves a task that requires participants to express or communicate feelings or emotions
   - as a core or critical element of the therapy
   - and that aims to improve some aspect of patient or carer wellbeing.
- **Articles published in the English language** (to prevent issues with intricacies of translation interfering with an effective definition of key terms).
- **The majority of the population of interest are adult participants (aged 18 and above):**
   - With a diagnosis of an advanced disease (e.g. end-stage organ failure or advanced/metastatic/incurable cancers), and/or being explicitly treated with a palliative intent OR
   - Family carers of patients at a palliative stage of a disease.
   - Based on previous related reviews(44) that indicated that few studies meet these criteria, samples which included >50% patients with advanced-stage disease will also be included.
- **All types of original research from within the peer-reviewed medical and nursing, psychological and social science literature will be included,** including RCTs, comparative studies (e.g. non-randomised experiments, before-and-after studies), qualitative studies, case studies, ethnographies and diary studies.
- **Peer-reviewed conference abstracts** of papers not published in full will also be included if they are sufficiently detailed.
- **Review articles** that discuss ED as a psychotherapeutic intervention and make explicit mention of its use in the palliative care setting (or in patients with advanced disease and/or their family carers), including: systematic reviews, meta-analyses, meta-syntheses, scoping reviews, narrative reviews, rapid reviews, critical reviews and integrative reviews, opinion pieces, commentaries and editorial reviews.

Exclusion criteria

For peer review only - http://bmjopen.bmj.com/site/about/guidelines.xhtml

The following resources will be excluded from data extraction and analysis:

- Studies with tasks that were not designed to be emotionally expressive, or which do not list ED (or similar) as a key feature of the intervention
- Non peer-reviewed sources (e.g. some book chapters and dissertations/theses); however, we will scan reference lists of relevant resources, and/or contact authors where appropriate

No date limits will be applied to the searches, in order to capture the breadth of ED therapy delivery beyond the introduction of the well-cited EW paradigm in 1986.(16)

Databases

The following electronic databases will be searched from database inception to March 2019: the Cumulative Index to Nursing and Allied Health Literature (CINAHL), Cochrane Central Register of Controlled Trials (CENTRAL), PsycINFO, Scopus, Web of Science and MEDLINE. We will also check the European Union Clinical Trials Register, clinicaltrials.gov, the European Association for Palliative Care (EAPC) and British Psychological Society (BPS) conference abstract proceedings for the last seven (2012-2018) and 17 years (2001 – 2018) respectively. Additionally, we will check the reference lists of relevant studies, review articles, book chapters and theses to identify further relevant citations. Finally, we will contact researchers who have expressed an interest in the field, via a research list compiled by the BPS, to ask if they are aware of any studies that may be relevant to this review. In case of uncertainty, authors of relevant studies will be approached to clarify whether studies meet the inclusion criteria for this review.

Search strategy

The search strategy is based on an earlier systematic review exploring EW as a psychotherapeutic intervention in patients with advanced disease.(44) The search terms have been expanded to capture ED-based therapies more broadly, as well as including terms for advanced disease and palliative care. See supplementary file 2 for an example search strategy used in the Ovid MEDLINE

For peer review only - http://bmjopen.bmj.com/site/about/guidelines.xhtml

database that will be modified for each database, utilising keywords, MeSH terms and Boolean operators as appropriate. As per Levac and colleagues'(64) recommendation for an iterative search strategy development process, we will review the search criteria throughout the screening process to update and expand the search if required.

Stage 3: Study selection

The research team will meet to discuss preliminary inclusion and exclusion criteria during the protocol development phase. At least two reviewers will independently screen citations for inclusion to full article review stage. Reviewers will meet at the beginning, midpoint and final stages of the abstract review process to discuss challenges and uncertainties related to study selection, and to refine the search strategy and inclusion criteria if needed. Full article review will also be carried out independently by two researchers for articles which meet the inclusion criteria, or have unclear relevance during the screening phase. Where disagreements arise around inclusion, a third reviewer will be consulted to resolve disputes.

Stage 4: Charting the data

The research team will collectively develop the data-charting form based on the variables most relevant to the research questions. The form will be piloted using five articles, and the process and data-fields discussed between the research team prior to conducting the full data extraction procedure. Following full data extraction, the data from each independent reviewer will be compared and any discrepancies discussed to achieve consistency between reviewers.

A preliminary data extraction framework has been developed, tailored to answer each of the pre-defined research questions. Along with basic bibliographic information, information will be extracted about the study design, patient population, intervention characteristics, intervention objectives, outcome measures, underlying theoretical frameworks, intervention efficacy and proposed rationale for efficacy.

For peer review only

Stage 5: Collating, summarising and reporting the results

As per the guidelines of Levac and colleagues,(64) this stage will be conducted in three phases:

1) Analysis: to include both descriptive, quantitative analysis (e.g. number of relevant studies within each intervention type; sample demographics) and qualitative thematic analysis (to explore how different ED-based interventions may be classified by format, objectives and/or other characteristics to inform the taxonomy)
2) Reporting the results of the analysis and producing the outcomes that refer to the study's research question(s)
3) Considering the meanings of the findings as they relate to the overall study purpose, and discussing the implications for future research, practice and/or policy

Thematic analysis will be applied to understand the core, defining characteristics of each ED-based intervention.(67) From this analysis, we will work to develop a taxonomy of ED-based interventions. Thematic analysis of the objectives, outcomes and mechanisms will also be conducted, to enable us to map them onto intervention types. Exploratory analysis of facilitators, barriers and efficacy of specific interventions will also be conducted. We will examine whether there are any specific types of intervention that appear in studies using robust designs (e.g. RCTs) to produce higher proportions of positive outcomes associated with specific outcome measures. We will also examine whether these patterns of efficacy are related to specific facilitators or barriers. The aim of this analysis will be to provide an indication of the reported efficacy and setting-specific requirements, with the intention of providing insights into the most useful direction for future work.

Results will be reported as tables, graphs and descriptive themes as appropriate. As well as reporting a taxonomy of ED-based interventions in the palliative care context, we will also discuss how it can be used to help guide future research into and implementation of ED-based psychotherapy in this setting.

For peer review only - http://bmjopen.bmj.com/site/about/guidelines.xhtml

Stage 6: Consultation

Although not mandated by the Arksey and O'Malley(65) framework, in the extension developed by Levac and colleagues'(64) consultation with key stakeholders who may provide insights beyond the literature is essential. The research team who contributed to the development of this protocol includes a range of key stakeholders who will be engaged throughout the review process (including a palliative care consultant, a psychiatrist and researchers with expertise in EW, palliative care research and systematic review processes). An advisory group will also be consulted throughout the review process, including protocol development, results analysis and development of resulting conclusions and recommendations. The group includes health psychologist (Dr Nick Troop), clinical psychologist (Dr Penny Rapaport), former patient carer (Mr Peter Buckle), and evidence synthesis methodologist and palliative care nurse (Dr Kate Flemming).

PATIENT AND PUBLIC INVOLVEMENT

As described in Stage 6 (Consultation), Mr Peter Buckle is a member of the advisory group. Peter has lived experience of caring for his wife throughout her terminal illness. He is a member of the Marie Curie Research Expert Voices Group, a group of volunteers with personal experiences of living with terminal illness who support Marie Curie's research activities. Peter's insights will be used to throughout the review process, including development of the research protocol, results analysis and dissemination.

ETHICS AND DISSEMINATION

We present a protocol for a comprehensive and rigorous scoping review of ED-based interventions used in patients and family carers in the palliative care setting. Ethics approval is not required since the study involves only secondary analysis of data that has already been collected. The results will be disseminated through traditional routes, including peer-reviewed journals, local and international conferences on palliative care and health psychology, and press releases, social media and blogs as

For peer review only - http://bmjopen.bmj.com/site/about/guidelines.xhtml

appropriate. Through effective dissemination, the results of the review should help to inform more effective development, study and review of ED-based therapies in this patient population.

DATA AVAILABILITY

This is a review of published or open access literature.

REFERENCES

1. Ryan T, Ingleton C, Gardiner C, Parker C, Gott M, Noble B. Symptom burden, palliative care need and predictors of physical and psychological discomfort in two UK hospitals. BMC Palliat Care [Internet]. 2013 Dec [cited 2019 Jan 10];12(1). Available from: http://bmcpalliatcare.biomedcentral.com/articles/10.1186/1472-684X-12-11

2. Adelbratt S, Strang P. Death anxiety in brain tumour patients and their spouses. Palliat Med Lond. 2000 Nov;14(6):499–507.

3. Kasl-Godley JE, King DA, Quill TE. Opportunities for psychologists in palliative care: Working with patients and families across the disease continuum. Am Psychol. 2014;69(4):364–76.

4. Andershed B, Harstäde CW. Next of kin's feelings of guilt and shame in end-of-life care. Contemp Nurse J Aust Nurs Prof Maleny. 2007 Dec;27(1):61–72.

5. World Health Organisation. Planning and implementing palliative care services: a guide for programme managers [Internet]. WHO Press; 2016 [cited 2019 Jan 8]. Available from: http://apps.who.int/iris/bitstream/handle/10665/250584/9789241565417-eng.pdf;jsessionid=706DE6863C5CF9BAA2D2BD0A177D9B6D?sequence=1

6. Okuyama T, Akechi T, Mackenzie L, Furukawa TA. Psychotherapy for depression among advanced, incurable cancer patients: A systematic review and meta-analysis. Cancer Treat Rev. 2017 May;56:16–27.

7. Fulton JJ, Newins AR, Porter LS, Ramos K. Psychotherapy Targeting Depression and Anxiety for Use in Palliative Care: A Meta-Analysis. J Palliat Med. 2018 Jul;21(7):1024–37.

8. von Blanckenburg P, Leppin N. Psychological interventions in palliative care: Curr Opin Psychiatry. 2018 Sep;31(5):389–95.

9. Russell C, Fountain A. Role of clinical psychology in UK hospices. BMJ Support Palliat Care. 2018 Oct 3;bmjspcare-2018-001594.

10. Price A, Hotopf M, Higginson IJ, Monroe B, Henderson M. Psychological services in hospices in the UK and Republic of Ireland. 2006;99:3.

11. Gardiner C, Ryan T, Gott M. What is the cost of palliative care in the UK? A systematic review. BMJ Support Palliat Care. 2018 Sep;8(3):250–7.

12. Hospice UK, Together for short lives. Commissioning and statutory funding arrangements for hospices in England Survey results 2015 [Internet]. 2015 [cited 2019 Jan 21]. Available from:

For peer review only - http://bmjopen.bmj.com/site/about/guidelines.xhtml

https://www.hospiceuk.org/docs/default-source/What-We-Offer/Commissioning-support/hospice-commissioning-and-funding-survey-results-2015.pdf?sfvrsn=2

13. National Institute for Clinical Excellence. Guidance on cancer services: improving supportive and palliative care for adults with adults the manual. London: National Institute for Clinical Excellence; 2004.

14. Atkin N, Vickerstaff V, Candy B. 'Worried to death': the assessment and management of anxiety in patients with advanced life-limiting disease, a national survey of palliative medicine physicians. BMC Palliat Care [Internet]. 2017 Dec 11 [cited 2019 Jan 3];16. Available from: https://www.ncbi.nlm.nih.gov/pmc/articles/PMC5724241/

15. Lepore SJ, Smyth JM. The writing cure: An overview. In: Lepore SJ, Smyth JM, editors. The writing cure: How expressive writing promotes health and emotional well-being [Internet]. Washington: American Psychological Association; 2002 [cited 2019 Jan 8]. p. 3–14. Available from: http://content.apa.org/books/10451-014

16. Pennebaker JW, Beall SK. Confronting a Traumatic Event: Toward an Understanding of Inhibition and Disease. 1986;95(3):274–81.

17. Frisina PG, Borod JC, Lepore SJ. A Meta-Analysis of the Effects of Written Emotional Disclosure on the Health Outcomes of Clinical Populations: J Nerv Ment Dis. 2004 Sep;192(9):629–34.

18. Smyth JM, Pennebaker JW. Exploring the boundary conditions of expressive writing: In search of the right recipe. Br J Health Psychol. 2008 Feb 1;13(1):1–7.

19. Nicholls S. Beyond Expressive Writing: Evolving Models of Developmental Creative Writing. J Health Psychol. 2009 Mar;14(2):171–80.

20. King LA. The Health Benefits of Writing about Life Goals. Pers Soc Psychol Bull. 2001 Jul;27(7):798–807.

21. Marlo H, Wagner MK. Expression of negative and positive events through writing: Implications for psychotherapy and health. Psychol Health. 1999 Mar;14(2):193–215.

22. Nyssen OP, Taylor SJ, Wong G, Steed E, Bourke L, Lord J, et al. Does therapeutic writing help people with long-term conditions? Systematic review, realist synthesis and economic considerations. Health Technol Assess. 2016 Apr;20(27):1–368.

23. Pennebaker JW. Writing About Emotional Experiences as a Therapeutic Process. Psychol Sci. 1997 May;8(3):162–6.

24. Davies EA. Why we need more poetry in palliative care. BMJ Support Palliat Care. 2018;

25. Baker FA, Stretton-Smith P, Clark IN, Tamplin J, Lee Y-EC. A Group Therapeutic Songwriting Intervention for Family Caregivers of People Living With Dementia: A Feasibility Study With Thematic Analysis. Front Med [Internet]. 2018 May 22 [cited 2019 Jan 8];5. Available from: https://www.frontiersin.org/article/10.3389/fmed.2018.00151/full

26. Robb M, Miller A. Supervisee Art-Based Disclosure in El Duende Process Painting. Art Ther. 2017 Oct 2;34(4):192–200.

For peer review only - http://bmjopen.bmj.com/site/about/guidelines.xhtml

27. Taubert M, Watts G, Boland J, Radbruch L. Palliative social media. BMJ Support Palliat Care. 2014 Mar;4(1):13–8.

28. Taylor J, Pagliari C. #Deathbedlive: the end-of-life trajectory, reflected in a cancer patient's tweets. BMC Palliat Care [Internet]. 2018 Dec [cited 2019 Jan 8];17(1). Available from: https://bmcpalliatcare.biomedcentral.com/articles/10.1186/s12904-018-0273-9

29. Wright J, Chung MC. Mastery or mystery? Therapeutic writing: A review of the literature. Br J Guid Couns. 2001 Aug;29(3):277–91.

30. Craig P, Dieppe P, Macintyre S, Michie S, Nazareth I, Petticrew M. Developing and evaluating complex interventions: Following considerable development in the field since 2006, MRC and NIHR have jointly commissioned an update of this guidance to be published in 2019. [Internet]. 2006 [cited 2019 Jan 8]. Available from: https://mrc.ukri.org/documents/pdf/complex-interventions-guidance/

31. Evans CJ, Harding R, Higginson IJ, on behalf of MORECare. 'Best practice' in developing and evaluating palliative and end-of-life care services: A meta-synthesis of research methods for the MORECare project. Palliat Med. 2013 Dec;27(10):885–98.

32. Pennebaker JW. Theories, Therapies, and Taxpayers: On the Complexities of the Expressive Writing Paradigm. Clin Psychol Sci Pract. 2004;11(2):138–42.

33. Sloan DM, Marx BP. Taking Pen to Hand: Evaluating Theories Underlying the Written Disclosure Paradigm. Clin Psychol Sci Pract. 2004;11(2):121–37.

34. Tausczik YR, Pennebaker JW. The Psychological Meaning of Words: LIWC and Computerized Text Analysis Methods. J Lang Soc Psychol. 2010 Mar;29(1):24–54.

35. Chung C, Pennebaker J. The Psychological Functions of Function Words. :18.

36. Pennebaker JW, Mehl MR, Niederhoffer KG. Psychological Aspects of Natural Language Use: Our Words, Our Selves. Annu Rev Psychol. 2003;54(1):547–77.

37. Czamanski-Cohen J, Weihs KL. The bodymind model: A platform for studying the mechanisms of change induced by art therapy. Arts Psychother. 2016 Nov;51:63–71.

38. Paterson C, Jones M, Rattray J, Lauder W, Nabi G. What is the mechanism effect that links social support to coping and psychological outcome within individuals affected by prostate cancer? Real time data collection using mobile technology. Eur J Oncol Nurs. 2016 Apr;21:126–33.

39. Lin H, Tov W, Qiu L. Emotional disclosure on social networking sites: The role of network structure and psychological needs. Comput Hum Behav. 2014 Dec;41:342–50.

40. Balon S, Rimé B. Lexical Profile of Emotional Disclosure in Socially Shared Versus Written Narratives. J Lang Soc Psychol. 2016 Sep;35(4):345–73.

41. Giese-Davis J, Koopman C, Butler LD, Classen C, Cordova M, Fobair P, et al. Change in emotion-regulation strategy for women with metastatic breast cancer following supportive-expressive group therapy. J Consult Clin Psychol. 2002;70(4):916–25.

For peer review only - http://bmjopen.bmj.com/site/about/guidelines.xhtml

42. Schwartz CE, David E. To everything there is a season: A written expression intervention for closure at the end of life. In: Lepore SJ, Smyth JM, editors. The writing cure: How expressive writing promotes health and emotional well-being [Internet]. Washington: American Psychological Association; 2002 [cited 2019 Jan 21]. p. 257–78. Available from: http://content.apa.org/books/10451-013

43. Frattaroli J. Experimental disclosure and its moderators: A meta-analysis. Psychol Bull. 2006;132(6):823–65.

44. Kupeli N, Chatzithedorou G, Troop N, McInnerney D, Stone P, Candy B. Expressive writing as a therapeutic intervention for people with advanced disease: A systematic review. BMC Palliat Care.

45. Laccetti M. Expressive writing in women with advanced breast cancer. Oncol Nurs Forum. 2007 Sep;34(5):1019–24.

46. Imrie S, Troop NA. A pilot study on the effects and feasibility of compassion-focused expressive writing in Day Hospice patients. Palliat Support Care. 2012 Jun;10(2):115–22.

47. Zhou C, Wu Y, An S, Li X. Effect of Expressive Writing Intervention on Health Outcomes in Breast Cancer Patients: A Systematic Review and Meta-Analysis of Randomized Controlled Trials. Ozakinci G, editor. PLOS ONE. 2015 Jul 7;10(7):e0131802.

48. Merz EL, Fox RS, Malcarne VL. Expressive writing interventions in cancer patients: a systematic review. Health Psychol Rev. 2014 Jul 3;8(3):339–61.

49. de Moor C, Sterner J, Hall M, Warneke C, Gilani Z, Amato R, et al. A pilot study of the effects of expressive writing on psychological and behavioral adjustment in patients enrolled in a Phase II trial of vaccine therapy for metastatic renal cell carcinoma. Health Psychol. 2002;21(6):615–9.

50. Henry EA, Schlegel RJ, Talley AE, Molix LA, Bettencourt BA. The Feasibility and Effectiveness of Expressive Writing for Rural and Urban Breast Cancer Survivors. Oncol Nurs Forum Pittsburgh. 2010 Nov;37(6):749–57.

51. Stanton AL, Danoff-Burg S, Sworowski LA, Collins CA, Branstetter AD, Rodriguez-Hanley A, et al. Randomized, Controlled Trial of Written Emotional Expression and Benefit Finding in Breast Cancer Patients. J Clin Oncol. 2002 Oct 15;20(20):4160–8.

52. Gellaitry G, Peters K, Bloomfield D, Horne R. Narrowing the gap: the effects of an expressive writing intervention on perceptions of actual and ideal emotional support in women who have completed treatment for early stage breast cancer. Psychooncology. 2010 Jan;19(1):77–84.

53. Rosenberg HJ, Rosenberg SD, Ernstoff MS, Wolford GL, Amdur RJ, Elshamy MR, et al. Expressive Disclosure and Health Outcomes in a Prostate Cancer Population. Int J Psychiatry Med. 2002 Mar;32(1):37–53.

54. Mosher CE, Duhamel KN, Lam J, Dickler M, Li Y, Massie MJ, et al. Randomised trial of expressive writing for distressed metastatic breast cancer patients. Psychol Health. 2012;27(1):88–100.

55. Zachariae R, O'Toole MS. The effect of expressive writing intervention on psychological and physical health outcomes in cancer patients-a systematic review and meta-analysis: Effects of expressive writing intervention in cancer patients. Psychooncology. 2015 Nov;24(11):1349–59.

For peer review only

For peer review only - http://bmjopen.bmj.com/site/about/guidelines.xhtml

56. Kissane DW, Grabsch B, Clarke DM, Smith GC, Love AW, Bloch S, et al. Supportive-expressive group therapy for women with metastatic breast cancer: survival and psychosocial outcome from a randomized controlled trial. Psycho-Oncology. 2007 Apr 1;16(4):277–86.

57. Clements-Cortes A. The use of music in facilitating emotional expression in the terminally ill. Am J Hosp Palliat Care. 2004 Aug;21(4):255–60.

58. Vesel T, Soham D. Music Therapy and Palliative Care: Systematic Review. J Pain Symptom Manage. 2018 Dec 1;56(6):e74.

59. Bradt J, Dileo C, Magill L, Teague A. Music interventions for improving psychological and physical outcomes in cancer patients. Cochrane Gynaecological, Neuro-oncology and Orphan Cancer Group, editor. Cochrane Database Syst Rev [Internet]. 2016 Aug 15 [cited 2019 Jan 22]; Available from: http://doi.wiley.com/10.1002/14651858.CD006911.pub3

60. Archer S, Buxton S, Sheffield D. The effect of creative psychological interventions on psychological outcomes for adult cancer patients: a systematic review of randomised controlled trials: CPIs for cancer patients: a review. Psychooncology. 2015 Jan;24(1):1–10.

61. Barak A, Leichtentritt RD. Creative Writing after Traumatic Loss: Towards a Generative Writing Approach. Br J Soc Work. 2016 May 6;bcw030.

62. Lichtenthal WG, Cruess DG. Effects of Directed Written Disclosure on Grief and Distress Symptoms Among Bereaved Individuals. Death Stud. 2010 Jun 7;34(6):475–99.

63. Armstrong M, Flemming K, Kupeli N, Stone P, Wilkinson S, Candy B. O25 Using qualitative synthesis data to inform interventions in palliative care: a multilevel approach. BMJ Open. 2019;9(Suppl 1):A1–27.

64. Levac D, Colquhoun H, O'Brien KK. Scoping studies: advancing the methodology. Implement Sci [Internet]. 2010 Dec [cited 2019 Jan 10];5(1). Available from: http://implementationscience.biomedcentral.com/articles/10.1186/1748-5908-5-69

65. Arksey H, O'Malley L. Scoping studies: towards a methodological framework. Int J Soc Res Methodol. 2005 Feb;8(1):19–32.

66. The Joanna Briggs Institute. The Joanna Briggs Institute Reviewers' Manual 2015: Methodology for JBI Scoping Reviews. The Joanna Briggs Institute; 2015.

67. Braun V, Clarke V. Using thematic analysis in psychology. Qual Res Psychol. 2006 Jan;3(2):77–101.

AUTHOR STATEMENT

DM contributed to the conception and design of the study, developed and tested the search strategy and inclusion criteria, and drafted the protocol. BC, NK, PS, KA and JC contributed to the conception and design of the protocol, specifically providing insight into the rationale, search strategy and

For peer review only - http://bmjopen.bmj.com/site/about/guidelines.xhtml

inclusion criteria refinement, and critical review of the manuscript for clarity and intellectual content.

ACKNOWLEDGEMENTS

The authors would like to thank all members of the advisory group for their thoughtful contributions to the study design: Mr Peter Buckle, Dr Kate Flemming, Dr Penny Rapaport and Dr Nick Troop.

For peer review only

FUNDING

The time spent on this review by author DM was supported by a Marie Curie and Economic and Social Research Council collaborative grant (grant number: ES/P000592/1). The time spent on the review by authors BC and PS comes from core funding of the research department by Marie Curie (grant number: MCCC-FPO-16-U) and an Alzheimer's Society (AS) funded fellowship awarded to NK (grant number: 399 AS-JF-17b-016). The time spent on the review by author KA comes from a grant provided by Faculty of Medicine, Prince of Songkla University, Thailand. The time spent on the review by author JC comes from a Marie Curie Chair's Grant (grant number: 509537). The work of all authors is also supported by the UCLH NIHR Biomedical Research Centre.

COMPETING INTERESTS STATEMENT

None declared.

LICENSING INFORMATION

This is an open access article distributed in accordance with the Creative Commons Attribution 4.0 Unported (CC BY 4.0) license, which permits others to copy, redistribute, remix, transform and build upon this work for any purpose, provided the original work is properly cited, a link to the licence is

For peer review only - http://bmjopen.bmj.com/site/about/guidelines.xhtml

given, and indication of whether changes were made.

See: https://creativecommons.org/licenses/by/4.0/.

For peer review only

Supplementary file 1. Preferred Reporting Items for Systematic reviews and Meta-Analyses extension for Scoping Reviews (PRISMA-ScR) Checklist

SECTION	ITEM	PRISMA-ScR CHECKLIST ITEM	REPORTED ON PAGE #
**TITLE**			
Title	1	Identify the report as a scoping review.	1,2
**ABSTRACT**			
Structured summary	2	Provide a structured summary that includes (as applicable): background, objectives, eligibility criteria, sources of evidence, charting methods, results, and conclusions that relate to the review questions and objectives.	3-4
**INTRODUCTION**			
Rationale	3	Describe the rationale for the review in the context of what is already known. Explain why the review questions/objectives lend themselves to a scoping review approach.	5-9
Objectives	4	Provide an explicit statement of the questions and objectives being addressed with reference to their key elements (e.g., population or participants, concepts, and context) or other relevant key elements used to conceptualize the review questions and/or objectives.	10,12
**METHODS**			
Protocol and registration	5	Indicate whether a review protocol exists; state if and where it can be accessed (e.g., a Web address); and if available, provide registration information, including the registration number.	N/A
Eligibility criteria	6	Specify characteristics of the sources of evidence used as eligibility criteria (e.g., years considered, language, and publication status), and provide a rationale.	12-14
Information sources*	7	Describe all information sources in the search (e.g., databases with dates of coverage and contact with authors to identify additional sources), as well as the date the most recent search was executed.	14
Search	8	Present the full electronic search strategy for at least 1 database, including any limits used, such that it could be repeated.	Supplementary file 2
Selection of sources of evidence†	9	State the process for selecting sources of evidence (i.e., screening and eligibility) included in the scoping review.	15
Data charting process‡	10	Describe the methods of charting data from the included sources of evidence (e.g., calibrated forms or forms that have been tested by the team before their use, and whether data charting was done independently or in duplicate) and any processes for obtaining and confirming data from investigators.	15
Data items	11	List and define all variables for which data were sought and any assumptions and simplifications made.	12-14
Critical appraisal of individual sources of evidence§	12	If done, provide a rationale for conducting a critical appraisal of included sources of evidence; describe the methods used and how this information was used in any data synthesis (if appropriate).	N/A

For peer review only - http://bmjopen.bmj.com/site/about/guidelines.xhtml

SECTION	ITEM	PRISMA-ScR CHECKLIST ITEM	REPORTED ON PAGE #
Synthesis of results	13	Describe the methods of handling and summarizing the data that were charted.	15-16
**RESULTS**			
Selection of sources of evidence	14	Give numbers of sources of evidence screened, assessed for eligibility, and included in the review, with reasons for exclusions at each stage, ideally using a flow diagram.	N/A
Characteristics of sources of evidence	15	For each source of evidence, present characteristics for which data were charted and provide the citations.	N/A
Critical appraisal within sources of evidence	16	If done, present data on critical appraisal of included sources of evidence (see item 12).	N/A
Results of individual sources of evidence	17	For each included source of evidence, present the relevant data that were charted that relate to the review questions and objectives.	N/A
Synthesis of results	18	Summarize and/or present the charting results as they relate to the review questions and objectives.	N/A
**DISCUSSION**			
Summary of evidence	19	Summarize the main results (including an overview of concepts, themes, and types of evidence available), link to the review questions and objectives, and consider the relevance to key groups.	N/A
Limitations	20	Discuss the limitations of the scoping review process.	N/A
Conclusions	21	Provide a general interpretation of the results with respect to the review questions and objectives, as well as potential implications and/or next steps.	N/A
**FUNDING**			
Funding	22	Describe sources of funding for the included sources of evidence, as well as sources of funding for the scoping review. Describe the role of the funders of the scoping review.	23

JBI = Joanna Briggs Institute; PRISMA-ScR = Preferred Reporting Items for Systematic reviews and Meta-Analyses extension for Scoping Reviews.

* Where *sources of evidence* (see second footnote) are compiled from, such as bibliographic databases, social media platforms, and Web sites.

† A more inclusive/heterogeneous term used to account for the different types of evidence or data sources (e.g., quantitative and/or qualitative research, expert opinion, and policy documents) that may be eligible in a scoping review as opposed to only studies. This is not to be confused with *information sources* (see first footnote).

‡ The frameworks by Arksey and O'Malley (6) and Levac and colleagues (7) and the JBI guidance (4, 5) refer to the process of data extraction in a scoping review as data charting.

§ The process of systematically examining research evidence to assess its validity, results, and relevance before using it to inform a decision. This term is used for items 12 and 19 instead of "risk of bias" (which is more applicable to systematic reviews of interventions) to include and acknowledge the various sources of evidence that may be used in a scoping review (e.g., quantitative and/or qualitative research, expert opinion, and policy document).

Adapted from: Tricco AC, Lillie E, Zarin W, O'Brien KK, Colquhoun H, Levac D, et al. PRISMA Extension for Scoping Reviews (PRISMA-ScR): Checklist and Explanation. Ann Intern Med. ;169:467–473. doi: 10.7326/M18-0850

For peer review only

Supplementary file 2. Search strategy for Ovid PsycINFO: 1806 to December 2018

1 exp Emotions/ (300490)
2 emotion* (382265)
3 feeling* (103050)
4 1 OR 2 OR 3 (598583)

****

5 Palliative Care/ (11016)
6 (palliat* or terminal* or endstage or hospice* or metasta* or (end adj3 life) or (care adj3 dying) or ((advanced or late or last or end or final) adj3 (stage* or phase*))).tw. (54673)
7 5 OR 6 (54847)

****

8 (disclos* or express* or communicat* or talk* or speak* or spoke* or writ* or draw* or sing*).mp. (1005983)

****

9 4 AND 7 AND 8 (2575)

****

10 Apply filter: Humans (2278)
11 Apply filter: Adulthood (18+ years) (1320)

For peer review only

BMJ Open

Emotional disclosure as a therapeutic intervention in palliative care: a scoping review protocol

Journal:	*BMJ Open*
Manuscript ID	bmjopen-2019-031046.R1
Article Type:	Protocol
Date Submitted by the Author:	03-Jul-2019
Complete List of Authors:	McInnerney, Daisy; University College London, Marie Curie Palliative Care Research Department<br>Kupeli, Nuriye; University College London, Marie Curie Palliative Care Research Department<br>Stone, Patrick; University College London, Marie Curie Palliative Care Research Unit<br>Anantapong, Kanthee; University College London, Marie Curie Palliative Care Research Department; Prince of Songkla University Faculty of Medicine, Department of Psychiatry<br>Chan, Justin; University College London, Marie Curie Palliative Care Research Department<br>Candy, Bridget; University College London, Marie Curie Palliative Care Research Department
**Primary Subject Heading**:	Palliative care
Secondary Subject Heading:	Mental health, Oncology, Qualitative research
Keywords:	Emotional disclosure, Expressive writing, Psychotherapy, Advanced disease, PALLIATIVE CARE

SCHOLARONE™
Manuscripts

TITLE

Emotional disclosure as a therapeutic intervention in palliative care: a scoping review protocol

AUTHORS

Daisy McInnerney[1] (corresponding author), Marie Curie Palliative Care Research Department, Division of Psychiatry, University College London, London, UK; ORCID ID: 0000-0002-8921-2215

Nuriye Kupeli, Marie Curie Palliative Care Research Department, Division of Psychiatry, University College London, London, UK; ORCID ID: 0000-0001-6511-412X; n.kupeli@ucl.ac.uk

Patrick Stone, Marie Curie Palliative Care Research Department, Division of Psychiatry, University College London, London, UK; ORCID ID: 0000-0002-5765-9047; p.stone@ucl.ac.uk

Kanthee Anantapong, Marie Curie Palliative Care Research Department, Division of Psychiatry, University College London, London, UK and Department of Psychiatry, Faculty of Medicine, Prince of Songkla University, Thailand; kanthee.anantapong.18@ucl.ac.uk

Justin Chan, Marie Curie Palliative Care Research Department, Division of Psychiatry, University College London, London, UK; ORCID ID: 0000-0003-4144-441X; uctvjec@ucl.ac.uk

Bridget Candy, Marie Curie Palliative Care Research Department, Division of Psychiatry, University College London, London, UK; b.candy@ucl.ac.uk

[1] Marie Curie Palliative Care Research Department, Division of Psychiatry, University College London, 6th Floor, Maple House, 149 Tottenham Court Road, London W1T 7NF; Email: daisy.mcinnerney.18@ucl.ac.uk; Phone: +44(0) 7833 296 035

For peer review only - http://bmjopen.bmj.com/site/about/guidelines.xhtml

WORD COUNT

3892

For peer review only - http://bmjopen.bmj.com/site/about/guidelines.xhtml

ABSTRACT

Introduction

Emotional disclosure (ED) is a term used to describe the psychotherapeutic expression of emotion. ED underlies a variety of therapies aimed at improving wellbeing for various populations, including people with palliative-stage disease and their family carers. Systematic reviews of ED-based psychotherapy have largely focused on expressive writing (EW) as a way of generating ED. However, heterogeneity in intervention format and outcome measures has made it difficult to analyse efficacy. There is also debate about the mechanisms proposed to explain the potential effects of ED.

We present a scoping review protocol to develop a taxonomy of ED-based interventions to identify and categorise the spectrum of interventions that could be classified under the umbrella term of ‘emotional disclosure’ in the palliative care setting. By mapping these to associated treatment objectives, outcome measures and explanatory frameworks, the review will inform future efforts to design and evaluate ED-based therapies in this population.

Methods and analysis

The review will be guided by Arksey and O’Malley’s five-stage scoping review framework and Levac’s extension. The following electronic databases will be searched from database inception: CENTRAL, CINAHL, PsycINFO, Scopus, Web of Science and MEDLINE. We will include peer-reviewed studies and reviews. We will also check grey literature, including clinical trial registers, conference proceedings and reference lists, as well as contacting researchers. Articles will be screened by at least two independent reviewers and data charted using an extraction form developed for this review. Results will be analysed thematically to create a taxonomy of interventions, outcome measures and theoretical frameworks.

Ethics and dissemination

For peer review only

This review does not require ethical approval as it is a secondary analysis of pre-existing, published data. The results will inform future research in the development of ED-based interventions and evaluation of their efficacy in the palliative care setting. We will disseminate findings through peer-reviewed journals.

ARTICLE SUMMARY

Strengths and limitations of this study

- Despite the heterogeneity of the intervention repeatedly being cited as a limiting factor in evaluations of intervention efficacy, no previous study that we are aware of has systematically mapped the variety of ED-based interventions, outcome measures and theoretical frameworks used in the palliative care setting.
- A rigorous, systematic approach will be applied to searching, screening, extracting and analysing the literature based on established scoping review methodological approaches.
- There may be challenges in identifying studies due to the differences in terms used to describe ED-based interventions.
- The review will be limited to studies of adult populations and published in the English language.
- The studies included in the review will not be appraised for methodological quality as this is outside the remit of scoping review methodology.

KEY WORDS

Emotional disclosure; expressive writing; psychotherapy; palliative care; advanced disease

For peer review only - http://bmjopen.bmj.com/site/about/guidelines.xhtml

INTRODUCTION

People living with a terminal illness often experience significant psychological, emotional and physical discomfort.(1,2) Family carers are also likely to experience psychological distress during and after supporting a relative through advanced illness.(2–4) Palliative care services aim to holistically address the physical, psychological and other needs of patients with advanced, life-limiting illness.(5) Psychotherapies form one important element of this palliative treatment approach.(6–8) However, access to such therapies in this setting is restricted by issues arising from limited availability of qualified professionals and evidence-based interventions well-adapted to the population.(9,10) Funding also poses a potential challenge, with a 2015 survey of UK hospices identifying significant concerns over freezing or reduction of statutory funding, and warning of its adverse effects on hospice services.(11,12) Furthermore, guidelines for the provision of psychological services in this population are limited. The National Institute of Health and Care Excellence (NICE) last published guidance on structuring psychological support in palliative care for adults in 2004 and only in cancer. It is not clear how widely these guidelines have been followed.(9,13) Indeed, a recent survey of clinical psychologists working in UK hospices indicated there is significant variability in how psychological support is provided.(9) Palliative care physicians also report limited access to psychological services for patients.(14) Taken together, this suggests that palliative care services might benefit from access to simple interventions that can be delivered by non-specialist health care practitioners, and with the potential for volunteer involvement. This could supplement the support offered by specialist practitioners to clinically distressed patients with complex needs.

Emotional disclosure (ED) is a term used to describe the therapeutic expression of emotions. This flexible therapy holds potential to be harnessed as a relatively low-cost, simple intervention in certain formats, such as expressive writing.(15) Its flexibility also means it could be adapted to the specific needs of patients at a palliative stage of disease, for example, by modifying the method used

For peer review only - http://bmjopen.bmj.com/site/about/guidelines.xhtml

to generate emotional expression (for example, typing or spoken disclosure). ED has long been a critical concept associated with the talk-based psychotherapies pioneered by Freud in the early 1900s.(15) However, rigorous research into the concept and associated therapies was relatively scant until the 1980s.(15) In 1986, Pennebaker and Beall introduced their influential Expressive Writing (EW) intervention, which was designed to generate ED and led to a rapid expansion of research into this topic.(15,16) EW typically involves participants writing about their emotions associated with a traumatic experience for 15-20 minutes over 3-5 consecutive days. Since the introduction of Pennebaker and Beall's EW intervention, the format has been widely adapted to explore the boundary conditions of the intervention.(16–19) For example, tasks to induce ED have included writing about positive emotions or future goals, or spoken disclosure.(18–23) Poetry therapy is also recognised as an adaptation of the original EW paradigm.(22,24) Moreover, ED is recognised as a fundamental part of other forms of psychotherapy, such as music therapy(25) and art therapy.(26) In recent times, patients and family members are increasingly turning to blogging, social media and chatroom sites to disclose emotions around their experiences of their illness.(27–29)

The existence of numerous formats of ED-based therapies and behaviours complicates the process of exploring if and how such interventions might work. Yet, a clear understanding of the mechanisms linking cause and effect is considered fundamental to the development and study of complex interventions.(30,31) In the case of ED, there is unlikely to be a single underlying process, rather a framework of interacting mechanisms.(19,32,33) Processes that have been proposed to explain EW have been reviewed and include emotional inhibition, cognitive adaptation, exposure and emotional regulation,(19,32,33) yet no consensus has been reached on a unifying framework. Different methods of generating ED are also likely to invoke different or overlapping processes. For instance, disclosure through EW may employ mechanisms related to language processing(34–36) and ED

For peer review only - http://bmjopen.bmj.com/site/about/guidelines.xhtml

For peer review only

through art may function through sensory and motor processes.(37) Disclosure via online forums is likely to involve many of the mechanisms involved in social support.(38–40) Moreover, the therapeutic setting may influence which cognitive processes are initiated by ED. For instance, for patients at the palliative stage of disease, the potential effects of ED may be mediated by mechanisms related to meaning, control or closure in ways that may not occur in a healthy population.(41,42)

Mirroring the unclear processes underlying ED, the efficacy of ED-based therapies remains uncertain. In general populations, a meta-analysis of 146 studies identified a small but significant positive effect of ED-based interventions on both physical and psychological health outcomes in healthy populations.(43) It has been suggested that moderators, such as demographic, personality, and existing emotional support, are also likely to influence the efficacy of ED-based therapies, highlighting the importance of targeting and tailoring such interventions.(43) In people receiving palliative care, evidence of efficacy also remains unclear. Much of the literature is focused on EW and reports mixed results. A recent systematic review and meta-analysis of four randomised controlled trials (RCTs) that examined EW in patients with advanced disease found it did not have a significant effect on any of the selected health-related outcomes.(44) However, the review also reported more promising results from studies that conducted linguistic analyses of EW in this population; these analyses identified that use of certain words, such as positive emotion words, was related to better emotional wellbeing.(45) They also found EW writers used more cognitive words associated with causal understanding, which the authors reported suggested cognitive changes.(46) Other related reviews have also uncovered mixed results. A systematic review of EW in patients with breast cancer (irrespective of stage) found the intervention reduced negative somatic symptoms at a 3-month follow-up, although it had no effect on psychological outcome measures.(47) Another review(48) assessing EW in a variety of cancers of all stages reported 6 of 13 studies as finding statistically significant, small to moderate benefits of EW on energy and sleep patterns,(49)

For peer review only - http://bmjopen.bmj.com/site/about/guidelines.xhtml

depressive(50) and physical symptoms,(50,51) emotional support,(52) pain,(53) uptake of mental health services,(54) and healthcare utilisation.(51) However, each of the 13 studies also reported some null effects, and the authors were unable to conduct a meta-analysis due to the heterogeneity of interventions and outcome measures used across the studies. A further review of EW in cancer populations found no evidence of EW efficacy on psychological, physical or quality of life outcomes,(55) whilst a review of therapeutic writing in patients with long-term illnesses also found no effects.(22) None of the studies of EW in populations with advanced disease reported significant negative effects, with the exception of Low et al. (2010), who found women who had been diagnosed with metastatic cancer for ≥ 4.7 years exhibited greater sleep disturbance following EW, whereas those more recently diagnosed did not.

Such mixed results are also characteristic of other ED-based interventions in clinical populations. A preliminary scoping of the literature indicates that reviews of studies of explicitly ED-based interventions in the palliative care setting are lacking. However, individual studies have identified some benefits in this population. Kissane et al. (2016) found supportive-expressive group therapy improved quality of life and treatment of depression in women with metastatic breast cancer.(56) Whilst Clements-Cortes (2004) found patients with a terminal illness were able to decrease depressive symptoms and social isolation and enhance relaxation by expressing their emotions through music therapy.(57) Indeed, a recent review of music therapy in patients receiving palliative care found it had a positive effect on pain, fatigue, anxiety and quality of life.(58) Given the heterogeneity of interventions included in the review, however, not all included interventions were necessarily designed specifically to evoke ED. Reviews of ED-based interventions in clinical populations with serious (although not explicitly palliative-stage) disease have identified mixed results. A systematic review of 52 trials reported that music interventions may have beneficial effects on anxiety, pain, fatigue and quality-of-life for people with cancer (of all stages), but that results were inconsistent across trials.(59) They also noted that, when asked, participants said they

For peer review only - http://bmjopen.bmj.com/site/about/guidelines.xhtml

valued the opportunity for emotional expression and processing offered by the therapy. A further systematic review of Creative Psychological Interventions (CPIs), which encompass the use of music, art, drama and dance/movement to express and process thoughts and emotions, found evidence of psychological but not physical benefits of CPIs across ten trials in cancer patients.(60) Similarly, ED-based interventions in bereaved family members have also demonstrated mixed results,(61,62) although they have been less widely studied in this group than in patients.

Whilst the efficacy of ED-based therapies in the palliative care setting remains uncertain, current reviews recommend further research to assess the true efficacy of each intervention.(22,44,47,48,55,59,60) This is due to a number of limitations of the current literature. First, current reviews are significantly limited by the heterogeneity of the format of interventions and outcome measures used across the studies they are reviewing.(22,44,47,48,55,59,60) Second, and tellingly, qualitative interviews show participants find certain interventions valuable, even where null effects are captured by quantitative measures.(48,59) This suggests current studies are not necessarily investigating the outcome measures that convey the benefit experienced by patients, which may be more abstract or existential in nature, particularly in patients with a palliative-stage disease.(48,63) Third, authors have noted a lack of effort to tailor interventions to the specific needs of people with advanced or terminal illness.(22,44,48) This could encompass, for example, offering audio recorded disclosure as an alternative to written disclosure in EW studies, as some patients may lose the ability to write. Finally, methodological quality of studies included in the systematic reviews has been largely graded as low, with limitations due to sample size, methodological features such as lack of randomisation and data reporting. In light of these significant limitations to existing research, future studies should aim to address these shortfalls. The broad nature of therapeutic ED, however, makes future research design challenging. This is due, in part, to the significant overlap of terms being used to describe various interventions, and a lack of clarity on the most appropriate format and outcome measures and underlying mechanisms.

For peer review only

To address these shortcomings, we plan to use scoping review methodology to conduct an explorative, yet systematic, investigation of the existing heterogeneous literature. Scoping review methodology can be used to clarify and map out complex concepts in a robust and replicable manner.(64–66) It is therefore a suitable method through which to identify, consolidate and categorise the existing literature into a taxonomy of emotional-disclosure based interventions in the palliative care setting. Such a taxonomy will provide researchers with a framework to inform the design of future studies of ED-based interventions, by guiding selection of intervention format and outcome measures. Moreover, the taxonomy will map intervention efficacy, along with any reported facilitators and barriers, to intervention format, to help draw out potential mechanisms of action. If researchers use the taxonomy to inform study design, this should in turn lay the groundwork for more informative systematic reviews of ED-based interventions. Given the unique physical, psychological and emotional position (e.g. in terms of needs and experiences) of patients at the palliative stage of disease and their family carers, the scope of this review will be limited to research conducted in the palliative care setting. However, these findings could also provide a springboard to help develop a taxonomy for ED-based interventions in other populations.

For peer review only

Objectives

Primary objective

To develop a taxonomy of ED-based interventions used in the palliative care setting, for people with advanced diseases and their family carers. The taxonomy will identify, categorise and define classes of intervention that fall under the umbrella term of ‘emotional disclosure’.

Secondary objective

For peer review only - http://bmjopen.bmj.com/site/about/guidelines.xhtml

To map classes of intervention defined in the taxonomy to (1) underlying mechanisms, (2) appropriate treatment objectives, (3) outcome measures, (4) any facilitators and barriers to intervention feasibility and (5) efficacy.

METHODS AND ANALYSIS

This protocol is guided by the standard framework proposed by Arksey and O'Malley(65) and expanded by Levac and colleagues(64) and the Joanna Briggs Institute.(66) These guidelines recommend organising the scoping review process into at least five stages, with an optional sixth stage:

- Stage 1. Identifying the research question(s) - complete
- Stage 2. Identifying relevant studies
- Stage 3. Study selection
- Stage 4. Charting the data
- Stage 5. Collating, summarising and reporting the results
- Stage 6. Consultation

The protocol has also been developed in line with scoping review best practice, as summarised in the completed Preferred Reporting Items for Systematic Reviews and Meta-Analyses for Protocols and Scoping Reviews (PRISMA-ScR) included in supplementary file 1. Table 1 summarises the proposed timescale for the review.

For peer review only - http://bmjopen.bmj.com/site/about/guidelines.xhtml

	Month (2019)									
	Jan	Feb	Mar	Apr	May	Jun	Jul	Aug	Sep	Oct →
**Stage 1.** Identifying research question and writing protocol	■									
**Stage 2.** Identifying relevant studies (search)	■	■	■							
**Stage 3.** Study selection (screening)			■	■						
**Stage 4.** Data charting				■	■	■				
**Stage 5.** Collating, summarising and reporting results							■	■	■	■
**Stage 6.** Consultation	*Throughout process at key stages (protocol development, screening, collating results)*									

Table 1. Timeline for protocol and scoping review

Stage 1: Identifying the research question

To meet the objectives of the review, as outlined above, we will seek to thematically analyse insights from the following research questions:

- Which psychotherapeutic interventions delivered in patients at the palliative stage of disease and their family carers are categorised as, or explicitly grounded in, principles of ED? E.g. What format are the interventions, how often are they delivered and by whom?
- What are the primary objectives of ED-based interventions delivered in this setting? For example, to enhance overall quality of life, physical or psychological health.
- What outcome measures are used to assess the efficacy of ED-based interventions in this setting?

For peer review only

For peer review only - http://bmjopen.bmj.com/site/about/guidelines.xhtml

- What theoretical frameworks are used to explain the mechanisms underlying ED-based interventions in this setting?
- What are the facilitators and barriers to ED-based intervention feasibility and efficacy in this setting?

Stage 2: Identifying relevant studies

The following inclusion criteria were developed in collaboration with key stakeholders, including physicians, psychologists and supportive care service managers involved in the provision of palliative care. Throughout the screening and data extraction process the criteria will be discussed within the research team and updated where necessary to ensure all relevant literature is being captured.(64)

- **Studies must use or make reference to a psychotherapeutic intervention that the authors state:**
  - involves 'emotional disclosure' or involves a task that requires participants to express or communicate feelings or emotions
  - as a core or critical element of the therapy
  - and that aims to improve some aspect of patient or carer wellbeing.
- **Articles published in the English language** (to prevent issues with intricacies of translation interfering with an effective definition of key terms).
- **The majority of the population of interest are adult participants (aged 18 and above):**
  - With a diagnosis of an advanced disease (e.g. end-stage organ failure or advanced/metastatic/incurable cancers), and/or being explicitly treated with a palliative intent OR
  - Family carers of patients at a palliative stage of a disease.

For peer review only - http://bmjopen.bmj.com/site/about/guidelines.xhtml

  - Based on previous related reviews(44) that indicated that few studies meet these criteria, samples which included >50% patients with advanced-stage disease will also be included.

- **All types of original research from within the peer-reviewed medical and nursing, psychological and social science literature will be included,** including RCTs, comparative studies (e.g. non-randomised experiments, before-and-after studies), qualitative studies, case studies, ethnographies and diary studies.
- **Peer-reviewed conference abstracts** of papers not published in full will also be included if they are sufficiently detailed.
- **Review articles** that discuss ED as a psychotherapeutic intervention and make explicit mention of its use in the palliative care setting (or in patients with advanced disease and/or their family carers), including: systematic reviews, meta-analyses, meta-syntheses, scoping reviews, narrative reviews, rapid reviews, critical reviews and integrative reviews, opinion pieces, commentaries and editorial reviews.

Exclusion criteria

The following resources will be excluded from data extraction and analysis:

- Studies with tasks that were not designed to be emotionally expressive, or which do not list ED (or similar) as a key feature of the intervention
- Non peer-reviewed sources (e.g. some book chapters and dissertations/theses); however, we will scan reference lists of relevant resources, and/or contact authors where appropriate

No date limits will be applied to the searches, in order to capture the breadth of ED therapy delivery beyond the introduction of the well-cited EW paradigm in 1986.(16)

Databases

For peer review only - http://bmjopen.bmj.com/site/about/guidelines.xhtml

The following electronic databases will be searched from database inception to March 2019: the Cumulative Index to Nursing and Allied Health Literature (CINAHL), Cochrane Central Register of Controlled Trials (CENTRAL), PsycINFO, Scopus, Web of Science and MEDLINE. We will also check the European Union Clinical Trials Register, clinicaltrials.gov, the European Association for Palliative Care (EAPC) and British Psychological Society (BPS) conference abstract proceedings for the last seven (2012-2018) and 17 years (2001 – 2018) respectively. Additionally, we will check the reference lists of relevant studies, review articles, book chapters and theses to identify further relevant citations. Finally, we will contact researchers who have expressed an interest in the field, via a research list compiled by the BPS, to ask if they are aware of any studies that may be relevant to this review. In case of uncertainty, authors of relevant studies will be approached to clarify whether studies meet the inclusion criteria for this review.

Search strategy

The search strategy is based on an earlier systematic review exploring EW as a psychotherapeutic intervention in patients with advanced disease.(44) The search terms have been expanded to capture ED-based therapies more broadly, as well as including terms for advanced disease and palliative care. See supplementary file 2 for an example search strategy used in the Ovid MEDLINE database that will be modified for each database, utilising keywords, MeSH terms and Boolean operators as appropriate. As per Levac and colleagues'(64) recommendation for an iterative search strategy development process, we will review the search criteria throughout the screening process to update, expand or limit the search if required.

Stage 3: Study selection

The research team will meet to discuss preliminary inclusion and exclusion criteria during the protocol development phase. At least two reviewers will independently screen citations for inclusion to full article review stage. Reviewers will meet at the beginning, midpoint and final stages of the abstract review process to discuss challenges and uncertainties related to study selection, and to

For peer review only - http://bmjopen.bmj.com/site/about/guidelines.xhtml

refine the search strategy and inclusion criteria if needed. Full article review will also be carried out independently by two researchers for articles which meet the inclusion criteria, or have unclear relevance during the screening phase. Where disagreements arise around inclusion, a third reviewer will be consulted to resolve disputes.

Stage 4: Charting the data

The research team will collectively develop the data-charting form based on the variables most relevant to the research questions. The form will be piloted using five articles, and the process and data-fields discussed between the research team prior to conducting the full data extraction procedure. Following full data extraction, the data from each independent reviewer will be compared and any discrepancies discussed to achieve consistency between reviewers.

A preliminary data extraction framework has been developed, tailored to answer each of the pre-defined research questions. Along with basic bibliographic information, information will be extracted about the study design, patient population, intervention characteristics, intervention objectives, outcome measures, underlying theoretical frameworks, intervention efficacy and proposed rationale for efficacy. A draft data-charting form for primary experimental studies is included in supplementary file 3.

Stage 5: Collating, summarising and reporting the results

As per the guidelines of Levac and colleagues,(64) this stage will be conducted in three phases:

1) Analysis: to include both descriptive, quantitative analysis (e.g. number of relevant studies within each intervention type; sample demographics) and qualitative thematic analysis (to explore how different ED-based interventions may be classified by format, objectives and/or other characteristics to inform the taxonomy)
2) Reporting the results of the analysis and producing the outcomes that refer to the study's research question(s)

For peer review only - http://bmjopen.bmj.com/site/about/guidelines.xhtml

3) Considering the meanings of the findings as they relate to the overall study purpose, and discussing the implications for future research, practice and/or policy

Thematic analysis will be applied to understand the core, defining characteristics of each ED-based intervention.(67) From this analysis, we will work to develop a taxonomy of ED-based interventions. Thematic analysis of the objectives, outcomes and mechanisms will also be conducted, to enable us to map them onto intervention types. Exploratory analysis of facilitators, barriers and efficacy of specific interventions will also be conducted. We will examine whether there are any specific types of intervention that appear in studies using robust designs (e.g. RCTs) to produce higher proportions of positive outcomes associated with specific outcome measures. We will also examine whether these patterns of efficacy are related to specific facilitators or barriers. The aim of this analysis will be to provide an indication of the reported efficacy and setting-specific requirements, with the intention of providing insights into the most useful direction for future work.

Results will be reported as tables, graphs and descriptive themes as appropriate. As well as reporting a taxonomy of ED-based interventions in the palliative care context, we will also discuss how it can be used to help guide future research into and implementation of ED-based psychotherapy in this setting.

Stage 6: Consultation

Although not mandated by the Arksey and O'Malley(65) framework, in the extension developed by Levac and colleagues'(64) consultation with key stakeholders who may provide insights beyond the literature is essential. The research team who contributed to the development of this protocol includes a range of key stakeholders who will be engaged throughout the review process (including a palliative care consultant, a psychiatrist and researchers with expertise in EW, palliative care research and systematic review processes). An advisory group will also be consulted throughout the review process, including protocol development, results analysis and development of resulting conclusions and recommendations. The group includes health psychologist (Dr Nick Troop), clinical

For peer review only - http://bmjopen.bmj.com/site/about/guidelines.xhtml

psychologist (Dr Penny Rapaport), former patient carer (Mr Peter Buckle), and evidence synthesis methodologist and palliative care nurse (Dr Kate Flemming).

PATIENT AND PUBLIC INVOLVEMENT

As described in Stage 6 (Consultation), Mr Peter Buckle is a member of the advisory group. Peter has lived experience of caring for his wife throughout her terminal illness. He is a member of the Marie Curie Research Expert Voices Group, a group of volunteers with personal experiences of living with terminal illness who support Marie Curie's research activities. Peter's insights will be used to throughout the review process, including development of the research protocol, results analysis and dissemination.

ETHICS AND DISSEMINATION

We present a protocol for a comprehensive and rigorous scoping review of ED-based interventions used in patients and family carers in the palliative care setting. Ethics approval is not required since the study involves only secondary analysis of data that has already been collected. The results will be disseminated through traditional routes, including peer-reviewed journals, local and international conferences on palliative care and health psychology, and press releases, social media and blogs as appropriate. Through effective dissemination, the results of the review should help to inform more effective development, study and review of ED-based therapies in this patient population.

DATA AVAILABILITY

This is a review of published or open access literature.

REFERENCES

1. Ryan T, Ingleton C, Gardiner C, Parker C, Gott M, Noble B. Symptom burden, palliative care need and predictors of physical and psychological discomfort in two UK hospitals. BMC Palliat Care [Internet]. 2013 Dec [cited 2019 Jan 10];12(1). Available from: http://bmcpalliatcare.biomedcentral.com/articles/10.1186/1472-684X-12-11

For peer review only - http://bmjopen.bmj.com/site/about/guidelines.xhtml

2. Adelbratt S, Strang P. Death anxiety in brain tumour patients and their spouses. Palliat Med Lond. 2000 Nov;14(6):499–507.

3. Kasl-Godley JE, King DA, Quill TE. Opportunities for psychologists in palliative care: Working with patients and families across the disease continuum. Am Psychol. 2014;69(4):364–76.

4. Andershed B, Harstäde CW. Next of kin's feelings of guilt and shame in end-of-life care. Contemp Nurse J Aust Nurs Prof Maleny. 2007 Dec;27(1):61–72.

5. World Health Organisation. Planning and implementing palliative care services: a guide for programme managers [Internet]. WHO Press; 2016 [cited 2019 Jan 8]. Available from: http://apps.who.int/iris/bitstream/handle/10665/250584/9789241565417-eng.pdf;jsessionid=706DE6863C5CF9BAA2D2BD0A177D9B6D?sequence=1

6. Okuyama T, Akechi T, Mackenzie L, Furukawa TA. Psychotherapy for depression among advanced, incurable cancer patients: A systematic review and meta-analysis. Cancer Treat Rev. 2017 May;56:16–27.

7. Fulton JJ, Newins AR, Porter LS, Ramos K. Psychotherapy Targeting Depression and Anxiety for Use in Palliative Care: A Meta-Analysis. J Palliat Med. 2018 Jul;21(7):1024–37.

8. von Blanckenburg P, Leppin N. Psychological interventions in palliative care: Curr Opin Psychiatry. 2018 Sep;31(5):389–95.

9. Russell C, Fountain A. Role of clinical psychology in UK hospices. BMJ Support Palliat Care. 2018 Oct 3;bmjspcare-2018-001594.

10. Price A, Hotopf M, Higginson IJ, Monroe B, Henderson M. Psychological services in hospices in the UK and Republic of Ireland. 2006;99:3.

11. Gardiner C, Ryan T, Gott M. What is the cost of palliative care in the UK? A systematic review. BMJ Support Palliat Care. 2018 Sep;8(3):250–7.

12. Hospice UK, Together for short lives. Commissioning and statutory funding arrangements for hospices in England Survey results 2015 [Internet]. 2015 [cited 2019 Jan 21]. Available from: https://www.hospiceuk.org/docs/default-source/What-We-Offer/Commissioning-support/hospice-commissioning-and-funding-survey-results-2015.pdf?sfvrsn=2

13. National Institute for Clinical Excellence. Guidance on cancer services: improving supportive and palliative care for adults with adults the manual. London: National Institute for Clinical Excellence; 2004.

14. Atkin N, Vickerstaff V, Candy B. 'Worried to death': the assessment and management of anxiety in patients with advanced life-limiting disease, a national survey of palliative medicine physicians. BMC Palliat Care [Internet]. 2017 Dec 11 [cited 2019 Jan 3];16. Available from: https://www.ncbi.nlm.nih.gov/pmc/articles/PMC5724241/

15. Lepore SJ, Smyth JM. The writing cure: An overview. In: Lepore SJ, Smyth JM, editors. The writing cure: How expressive writing promotes health and emotional well-being [Internet]. Washington: American Psychological Association; 2002 [cited 2019 Jan 8]. p. 3–14. Available from: http://content.apa.org/books/10451-014

For peer review only

For peer review only - http://bmjopen.bmj.com/site/about/guidelines.xhtml

16. Pennebaker JW, Beall SK. Confronting a Traumatic Event: Toward an Understanding of Inhibition and Disease. 1986;95(3):274–81.

17. Frisina PG, Borod JC, Lepore SJ. A Meta-Analysis of the Effects of Written Emotional Disclosure on the Health Outcomes of Clinical Populations: J Nerv Ment Dis. 2004 Sep;192(9):629–34.

18. Smyth JM, Pennebaker JW. Exploring the boundary conditions of expressive writing: In search of the right recipe. Br J Health Psychol. 2008 Feb 1;13(1):1–7.

19. Nicholls S. Beyond Expressive Writing: Evolving Models of Developmental Creative Writing. J Health Psychol. 2009 Mar;14(2):171–80.

20. King LA. The Health Benefits of Writing about Life Goals. Pers Soc Psychol Bull. 2001 Jul;27(7):798–807.

21. Marlo H, Wagner MK. Expression of negative and positive events through writing: Implications for psychotherapy and health. Psychol Health. 1999 Mar;14(2):193–215.

22. Nyssen OP, Taylor SJ, Wong G, Steed E, Bourke L, Lord J, et al. Does therapeutic writing help people with long-term conditions? Systematic review, realist synthesis and economic considerations. Health Technol Assess. 2016 Apr;20(27):1–368.

23. Pennebaker JW. Writing About Emotional Experiences as a Therapeutic Process. Psychol Sci. 1997 May;8(3):162–6.

24. Davies EA. Why we need more poetry in palliative care. BMJ Support Palliat Care. 2018;

25. Baker FA, Stretton-Smith P, Clark IN, Tamplin J, Lee Y-EC. A Group Therapeutic Songwriting Intervention for Family Caregivers of People Living With Dementia: A Feasibility Study With Thematic Analysis. Front Med [Internet]. 2018 May 22 [cited 2019 Jan 8];5. Available from: https://www.frontiersin.org/article/10.3389/fmed.2018.00151/full

26. Robb M, Miller A. Supervisee Art-Based Disclosure in El Duende Process Painting. Art Ther. 2017 Oct 2;34(4):192–200.

27. Taubert M, Watts G, Boland J, Radbruch L. Palliative social media. BMJ Support Palliat Care. 2014 Mar;4(1):13–8.

28. Taylor J, Pagliari C. #Deathbedlive: the end-of-life trajectory, reflected in a cancer patient's tweets. BMC Palliat Care [Internet]. 2018 Dec [cited 2019 Jan 8];17(1). Available from: https://bmcpalliatcare.biomedcentral.com/articles/10.1186/s12904-018-0273-9

29. Wright J, Chung MC. Mastery or mystery? Therapeutic writing: A review of the literature. Br J Guid Couns. 2001 Aug;29(3):277–91.

30. Craig P, Dieppe P, Macintyre S, Michie S, Nazareth I, Petticrew M. Developing and evaluating complex interventions: Following considerable development in the field since 2006, MRC and NIHR have jointly commissioned an update of this guidance to be published in 2019. [Internet]. 2006 [cited 2019 Jan 8]. Available from: https://mrc.ukri.org/documents/pdf/complex-interventions-guidance/

For peer review only - http://bmjopen.bmj.com/site/about/guidelines.xhtml

31. Evans CJ, Harding R, Higginson IJ, on behalf of MORECare. 'Best practice' in developing and evaluating palliative and end-of-life care services: A meta-synthesis of research methods for the MORECare project. Palliat Med. 2013 Dec;27(10):885–98.

32. Pennebaker JW. Theories, Therapies, and Taxpayers: On the Complexities of the Expressive Writing Paradigm. Clin Psychol Sci Pract. 2004;11(2):138–42.

33. Sloan DM, Marx BP. Taking Pen to Hand: Evaluating Theories Underlying the Written Disclosure Paradigm. Clin Psychol Sci Pract. 2004;11(2):121–37.

34. Tausczik YR, Pennebaker JW. The Psychological Meaning of Words: LIWC and Computerized Text Analysis Methods. J Lang Soc Psychol. 2010 Mar;29(1):24–54.

35. Chung C, Pennebaker J. The Psychological Functions of Function Words. :18.

36. Pennebaker JW, Mehl MR, Niederhoffer KG. Psychological Aspects of Natural Language Use: Our Words, Our Selves. Annu Rev Psychol. 2003;54(1):547–77.

37. Czamanski-Cohen J, Weihs KL. The bodymind model: A platform for studying the mechanisms of change induced by art therapy. Arts Psychother. 2016 Nov;51:63–71.

38. Paterson C, Jones M, Rattray J, Lauder W, Nabi G. What is the mechanism effect that links social support to coping and psychological outcome within individuals affected by prostate cancer? Real time data collection using mobile technology. Eur J Oncol Nurs. 2016 Apr;21:126–33.

39. Lin H, Tov W, Qiu L. Emotional disclosure on social networking sites: The role of network structure and psychological needs. Comput Hum Behav. 2014 Dec;41:342–50.

40. Balon S, Rimé B. Lexical Profile of Emotional Disclosure in Socially Shared Versus Written Narratives. J Lang Soc Psychol. 2016 Sep;35(4):345–73.

41. Giese-Davis J, Koopman C, Butler LD, Classen C, Cordova M, Fobair P, et al. Change in emotion-regulation strategy for women with metastatic breast cancer following supportive-expressive group therapy. J Consult Clin Psychol. 2002;70(4):916–25.

42. Schwartz CE, David E. To everything there is a season: A written expression intervention for closure at the end of life. In: Lepore SJ, Smyth JM, editors. The writing cure: How expressive writing promotes health and emotional well-being [Internet]. Washington: American Psychological Association; 2002 [cited 2019 Jan 21]. p. 257–78. Available from: http://content.apa.org/books/10451-013

43. Frattaroli J. Experimental disclosure and its moderators: A meta-analysis. Psychol Bull. 2006;132(6):823–65.

44. Kupeli N, Chatzithedorou G, Troop N, McInnerney D, Stone P, Candy B. Expressive writing as a therapeutic intervention for people with advanced disease: A systematic review. BMC Palliat Care.

45. Laccetti M. Expressive writing in women with advanced breast cancer. Oncol Nurs Forum. 2007 Sep;34(5):1019–24.

For peer review only - http://bmjopen.bmj.com/site/about/guidelines.xhtml

46. Imrie S, Troop NA. A pilot study on the effects and feasibility of compassion-focused expressive writing in Day Hospice patients. Palliat Support Care. 2012 Jun;10(2):115–22.

47. Zhou C, Wu Y, An S, Li X. Effect of Expressive Writing Intervention on Health Outcomes in Breast Cancer Patients: A Systematic Review and Meta-Analysis of Randomized Controlled Trials. Ozakinci G, editor. PLOS ONE. 2015 Jul 7;10(7):e0131802.

48. Merz EL, Fox RS, Malcarne VL. Expressive writing interventions in cancer patients: a systematic review. Health Psychol Rev. 2014 Jul 3;8(3):339–61.

49. de Moor C, Sterner J, Hall M, Warneke C, Gilani Z, Amato R, et al. A pilot study of the effects of expressive writing on psychological and behavioral adjustment in patients enrolled in a Phase II trial of vaccine therapy for metastatic renal cell carcinoma. Health Psychol. 2002;21(6):615–9.

50. Henry EA, Schlegel RJ, Talley AE, Molix LA, Bettencourt BA. The Feasibility and Effectiveness of Expressive Writing for Rural and Urban Breast Cancer Survivors. Oncol Nurs Forum Pittsburgh. 2010 Nov;37(6):749–57.

51. Stanton AL, Danoff-Burg S, Sworowski LA, Collins CA, Branstetter AD, Rodriguez-Hanley A, et al. Randomized, Controlled Trial of Written Emotional Expression and Benefit Finding in Breast Cancer Patients. J Clin Oncol. 2002 Oct 15;20(20):4160–8.

52. Gellaitry G, Peters K, Bloomfield D, Horne R. Narrowing the gap: the effects of an expressive writing intervention on perceptions of actual and ideal emotional support in women who have completed treatment for early stage breast cancer. Psychooncology. 2010 Jan;19(1):77–84.

53. Rosenberg HJ, Rosenberg SD, Ernstoff MS, Wolford GL, Amdur RJ, Elshamy MR, et al. Expressive Disclosure and Health Outcomes in a Prostate Cancer Population. Int J Psychiatry Med. 2002 Mar;32(1):37–53.

54. Mosher CE, Duhamel KN, Lam J, Dickler M, Li Y, Massie MJ, et al. Randomised trial of expressive writing for distressed metastatic breast cancer patients. Psychol Health. 2012;27(1):88–100.

55. Zachariae R, O'Toole MS. The effect of expressive writing intervention on psychological and physical health outcomes in cancer patients-a systematic review and meta-analysis: Effects of expressive writing intervention in cancer patients. Psychooncology. 2015 Nov;24(11):1349–59.

56. Kissane DW, Grabsch B, Clarke DM, Smith GC, Love AW, Bloch S, et al. Supportive-expressive group therapy for women with metastatic breast cancer: survival and psychosocial outcome from a randomized controlled trial. Psycho-Oncology. 2007 Apr 1;16(4):277–86.

57. Clements-Cortes A. The use of music in facilitating emotional expression in the terminally ill. Am J Hosp Palliat Care. 2004 Aug;21(4):255–60.

58. Vesel T, Soham D. Music Therapy and Palliative Care: Systematic Review. J Pain Symptom Manage. 2018 Dec 1;56(6):e74.

59. Bradt J, Dileo C, Magill L, Teague A. Music interventions for improving psychological and physical outcomes in cancer patients. Cochrane Gynaecological, Neuro-oncology and Orphan Cancer Group, editor. Cochrane Database Syst Rev [Internet]. 2016 Aug 15 [cited 2019 Jan 22]; Available from: http://doi.wiley.com/10.1002/14651858.CD006911.pub3

For peer review only - http://bmjopen.bmj.com/site/about/guidelines.xhtml

60. Archer S, Buxton S, Sheffield D. The effect of creative psychological interventions on psychological outcomes for adult cancer patients: a systematic review of randomised controlled trials: CPIs for cancer patients: a review. Psychooncology. 2015 Jan;24(1):1–10.

61. Barak A, Leichtentritt RD. Creative Writing after Traumatic Loss: Towards a Generative Writing Approach. Br J Soc Work. 2016 May 6;bcw030.

62. Lichtenthal WG, Cruess DG. Effects of Directed Written Disclosure on Grief and Distress Symptoms Among Bereaved Individuals. Death Stud. 2010 Jun 7;34(6):475–99.

63. Armstrong M, Flemming K, Kupeli N, Stone P, Wilkinson S, Candy B. O25 Using qualitative synthesis data to inform interventions in palliative care: a multilevel approach. BMJ Open. 2019;9(Suppl 1):A1–27.

64. Levac D, Colquhoun H, O'Brien KK. Scoping studies: advancing the methodology. Implement Sci [Internet]. 2010 Dec [cited 2019 Jan 10];5(1). Available from: http://implementationscience.biomedcentral.com/articles/10.1186/1748-5908-5-69

65. Arksey H, O'Malley L. Scoping studies: towards a methodological framework. Int J Soc Res Methodol. 2005 Feb;8(1):19–32.

66. The Joanna Briggs Institute. The Joanna Briggs Institute Reviewers' Manual 2015: Methodology for JBI Scoping Reviews. The Joanna Briggs Institute; 2015.

67. Braun V, Clarke V. Using thematic analysis in psychology. Qual Res Psychol. 2006 Jan;3(2):77–101.

AUTHOR STATEMENT

DM contributed to the conception and design of the study, developed and tested the search strategy and inclusion criteria, and drafted the protocol. BC, NK, PS, KA and JC contributed to the conception and design of the protocol, specifically providing insight into the rationale, search strategy and inclusion criteria refinement, and critical review of the manuscript for clarity and intellectual content.

ACKNOWLEDGEMENTS

The authors would like to thank all members of the advisory group for their thoughtful contributions to the study design: Mr Peter Buckle, Dr Kate Flemming, Dr Penny Rapaport and Dr Nick Troop.

For peer review only

For peer review only - http://bmjopen.bmj.com/site/about/guidelines.xhtml

FUNDING

The time spent on this review by author DM was supported by a Marie Curie and Economic and Social Research Council collaborative grant (grant number: ES/P000592/1). The time spent on the review by authors BC and PS comes from core funding of the research department by Marie Curie (grant number: MCCC-FPO-16-U) and an Alzheimer's Society (AS) funded fellowship awarded to NK (grant number: 399 AS-JF-17b-016). The time spent on the review by author KA comes from a grant provided by Faculty of Medicine, Prince of Songkla University, Thailand. The time spent on the review by author JC comes from a Marie Curie Chair's Grant (grant number: 509537). The work of all authors is also supported by the UCLH NIHR Biomedical Research Centre.

COMPETING INTERESTS STATEMENT

None declared.

LICENSING INFORMATION

This is an open access article distributed in accordance with the Creative Commons Attribution 4.0 Unported (CC BY 4.0) license, which permits others to copy, redistribute, remix, transform and build upon this work for any purpose, provided the original work is properly cited, a link to the licence is given, and indication of whether changes were made.

See: https://creativecommons.org/licenses/by/4.0/.

For peer review only

For peer review only - http://bmjopen.bmj.com/site/about/guidelines.xhtml

Supplementary file 1. Preferred Reporting Items for Systematic reviews and Meta-Analyses extension for Scoping Reviews (PRISMA-ScR) Checklist

SECTION	ITEM	PRISMA-ScR CHECKLIST ITEM	REPORTED ON PAGE #
**TITLE**			
Title	1	Identify the report as a scoping review.	1,2
**ABSTRACT**			
Structured summary	2	Provide a structured summary that includes (as applicable): background, objectives, eligibility criteria, sources of evidence, charting methods, results, and conclusions that relate to the review questions and objectives.	3-4
**INTRODUCTION**			
Rationale	3	Describe the rationale for the review in the context of what is already known. Explain why the review questions/objectives lend themselves to a scoping review approach.	5-9
Objectives	4	Provide an explicit statement of the questions and objectives being addressed with reference to their key elements (e.g., population or participants, concepts, and context) or other relevant key elements used to conceptualize the review questions and/or objectives.	10,12
**METHODS**			
Protocol and registration	5	Indicate whether a review protocol exists; state if and where it can be accessed (e.g., a Web address); and if available, provide registration information, including the registration number.	N/A
Eligibility criteria	6	Specify characteristics of the sources of evidence used as eligibility criteria (e.g., years considered, language, and publication status), and provide a rationale.	12-14
Information sources*	7	Describe all information sources in the search (e.g., databases with dates of coverage and contact with authors to identify additional sources), as well as the date the most recent search was executed.	14
Search	8	Present the full electronic search strategy for at least 1 database, including any limits used, such that it could be repeated.	Supplementary file 2
Selection of sources of evidence†	9	State the process for selecting sources of evidence (i.e., screening and eligibility) included in the scoping review.	15
Data charting process‡	10	Describe the methods of charting data from the included sources of evidence (e.g., calibrated forms or forms that have been tested by the team before their use, and whether data charting was done independently or in duplicate) and any processes for obtaining and confirming data from investigators.	15
Data items	11	List and define all variables for which data were sought and any assumptions and simplifications made.	12-14
Critical appraisal of individual sources of evidence§	12	If done, provide a rationale for conducting a critical appraisal of included sources of evidence; describe the methods used and how this information was used in any data synthesis (if appropriate).	N/A

For peer review only - http://bmjopen.bmj.com/site/about/guidelines.xhtml

SECTION	ITEM	PRISMA-ScR CHECKLIST ITEM	REPORTED ON PAGE #
Synthesis of results	13	Describe the methods of handling and summarizing the data that were charted.	15-16
**RESULTS**			
Selection of sources of evidence	14	Give numbers of sources of evidence screened, assessed for eligibility, and included in the review, with reasons for exclusions at each stage, ideally using a flow diagram.	N/A
Characteristics of sources of evidence	15	For each source of evidence, present characteristics for which data were charted and provide the citations.	N/A
Critical appraisal within sources of evidence	16	If done, present data on critical appraisal of included sources of evidence (see item 12).	N/A
Results of individual sources of evidence	17	For each included source of evidence, present the relevant data that were charted that relate to the review questions and objectives.	N/A
Synthesis of results	18	Summarize and/or present the charting results as they relate to the review questions and objectives.	N/A
**DISCUSSION**			
Summary of evidence	19	Summarize the main results (including an overview of concepts, themes, and types of evidence available), link to the review questions and objectives, and consider the relevance to key groups.	N/A
Limitations	20	Discuss the limitations of the scoping review process.	N/A
Conclusions	21	Provide a general interpretation of the results with respect to the review questions and objectives, as well as potential implications and/or next steps.	N/A
**FUNDING**			
Funding	22	Describe sources of funding for the included sources of evidence, as well as sources of funding for the scoping review. Describe the role of the funders of the scoping review.	23

JBI = Joanna Briggs Institute; PRISMA-ScR = Preferred Reporting Items for Systematic reviews and Meta-Analyses extension for Scoping Reviews.
* Where *sources of evidence* (see second footnote) are compiled from, such as bibliographic databases, social media platforms, and Web sites.
† A more inclusive/heterogeneous term used to account for the different types of evidence or data sources (e.g., quantitative and/or qualitative research, expert opinion, and policy documents) that may be eligible in a scoping review as opposed to only studies. This is not to be confused with *information sources* (see first footnote).
‡ The frameworks by Arksey and O'Malley (6) and Levac and colleagues (7) and the JBI guidance (4, 5) refer to the process of data extraction in a scoping review as data charting.
§ The process of systematically examining research evidence to assess its validity, results, and relevance before using it to inform a decision. This term is used for items 12 and 19 instead of "risk of bias" (which is more applicable to systematic reviews of interventions) to include and acknowledge the various sources of evidence that may be used in a scoping review (e.g., quantitative and/or qualitative research, expert opinion, and policy document).

Adapted from: Tricco AC, Lillie E, Zarin W, O'Brien KK, Colquhoun H, Levac D, et al. PRISMA Extension for Scoping Reviews (PRISMA-ScR): Checklist and Explanation. Ann Intern Med. ;169:467–473. doi: 10.7326/M18-0850

For peer review only

Supplementary file 2. Search strategy for Ovid PsycINFO: 1806 to 2019

1 exp Emotions/ (300490)
2 emotion* (382265)
3 feeling* (103050)
4 1 OR 2 OR 3 (598583)

****

5 Palliative Care/ (11016)
6 (palliat* or terminal* or endstage or hospice* or metasta* or (end adj3 life) or (care adj3 dying) or ((advanced or late or last or end or final) adj3 (stage* or phase*))).tw. (54673)
7 5 OR 6 (54847)

****

8 (disclos* or express* or communicat* or talk* or speak* or spoke* or writ* or draw* or sing*).mp. (1005983)

****

9 4 AND 7 AND 8 (2575)

****

10 Apply filter: Humans (2278)
11 Apply filter: Adulthood (18+ years) (1320)

For peer review only

Supplementary file 3. Data charting extraction categories

Citation (Authors, year, title, journal, volume, page):

Keywords:

1. Population

a. Country:
b. Size (n):
    a. Total
    b. Intervention
    c. Control group(s)
c. Disease and stage
d. Age
e. Sex
f. Ethnicity
g. Other of note
h. Patient healthcare setting (hospice, hospital etc.)

2. Study details

i. Study design
j. Study objective

3. Intervention details

k. Intervention name
l. Objective of intervention
m. Task description
n. Control task description
o. Number of task sessions total
p. Task schedule (days between tasks)
q. Intervention setting (where task is completed)

4. Outcome measures

r. Primary
s. Secondary
t. Other measures (covariates, moderators)
u. Assessment time-points

5. Results

v. Primary outcome results
w. Secondary outcome results
x. Other results

6. Setting specific intervention details

y. Explicit adaptations to the intervention
z. Facilitators and barriers to intervention feasibility

7. Theoretical frameworks described

For peer review only
